# Collagen 2A Type B Induction after 3D Bioprinting Chondrocytes *In Situ* into Osteoarthritic Chondral Tibial Lesion

**DOI:** 10.1177/1947603520903788

**Published:** 2020-02-18

**Authors:** Birgitta Gatenholm, Carl Lindahl, Mats Brittberg, Stina Simonsson

**Affiliations:** 1 Department of Orthopaedics, Institute of Clinical Sciences, the Sahlgrenska Academy at the University of Gothenburg, Gothenburg, Sweden; 2 Sahlgrenska University Hospital, Mölndal, Sweden; 3 Institute of Biomedicine, Department of Clinical Chemistry and Transfusion Medicine, University of Gothenburg, Gothenburg, Sweden; 4 Cartilage Repair Unit, University of Gothenburg, Region Halland Orthopaedics, Kungsbacka Hospital, Kungsbacka, Sweden

**Keywords:** osteoarthritis, 3D bioprinting, chondrocytes, 3D CAD model, cartilage

## Abstract

**Objective:**

Large cartilage defects and osteoarthritis (OA) cause cartilage loss and remain a therapeutic challenge. Three-dimensional (3D) bioprinting with autologous cells using a computer-aided design (CAD) model generated from 3D imaging has the potential to reconstruct patient-specific features that match an articular joint lesion.

**Design:**

To scan a human OA tibial plateau with a cartilage defect, retrieved after total knee arthroplasty, following clinical imaging techniques were used: (1) computed tomography (CT), (2) magnetic resonance imaging (MRI), and (3) a 3D scanner. From such a scan, a CAD file was obtained to generate G-code to control 3D bioprinting *in situ* directly into the tibial plateau lesion.

**Results:**

Highest resolution was obtained using the 3D scanner (2.77 times more points/mm^2^ than CT), and of the 3 devices tested, only the 3D scanner was able to detect the actual OA defect area. Human chondrocytes included in 3D bioprinted constructs produced extracellular matrix and formed cartilage tissue fragments after 2 weeks of differentiation and high levels of a mature splice version of collagen type II (Col IIA type B), characteristic of native articular cartilage and aggrecan (ACAN). Chondrocytes had a mean viability of 81% in prints after day 5 of differentiation toward cartilage and similar viability was detected in control 3D pellet differentiation of chondrocytes (mean viability 72%).

**Conclusion:**

Articular cartilage can be formed in 3D bioprints. Thus, this 3D bioprinting system with chondrocytes simulating a patient-specific 3D model provides an attractive strategy for future treatments of cartilage defects or early OA.

## Introduction

Cartilage has a limited ability to heal due to the limited capacity of mature chondrocytes to proliferate, immobility of chondrocytes and absence of a vascular network. Furthermore, patients with knee osteoarthritis (OA) suffer a continuous cartilage degradation process.^1,2^ Articular joint injuries and articular cartilage degeneration are associated with pain, disability, and huge socioeconomic costs.^3^ Microfracturing, implantation of osteochondral auto- and allografts, and autologous chondrocyte implantation, have previously been developed to repair and reconstruct damaged cartilage.^4-6^ Although local chondral lesions can potentially be treated successfully with, for example, cell therapies, large defects, and OA lesions remain immense challenges. Scaffold materials for tissue engineering in combination with cells have been proposed as an approach to repair bone and cartilage defects.^7^ Three-dimensional (3D) bioprinting is an additional manufacturing technique by which the cells and supporting biomaterial can be deposited layer-by-layer in an exact position to mimic the tissue architecture and allow the construction of specific implantation tailored to the patient based on medical imaging data.^8^ For this purpose, different medical imaging techniques can be used, such as magnetic resonance imaging (MRI), computer tomography (CT), and other 3D scanning techniques for 3D reconstruction of the defect site.^9^ To achieve an anatomical 3D reconstruction of the defect site with high resolution, obtaining the exact shape that fits the damaged area is critical for treatment. 3D scanning has previously been studied to obtain a precise 3D digital model of an artificially created defect in a pig model, which was subsequently filled in by 3D bioprinting *in situ* using hydrogels.^9^ To our knowledge, no OA defect in human cartilage has yet been scanned and subsequently used to 3D bioprint a perfect fit directly into the cartilage lesion area. In addition, the 3D scanner technique has been suggested to be better than MRI or CT but not actually been evaluated.^9^ Three-dimensional bioprinting technology is attractive in regenerative medicine because it can enable tissues and organs to be printed on demand, biofabricate very large constructs and be mass-produced. Recent reviews have summarized new directions in articular cartilage tissue engineering using 3D bioprinting, including subject-specific geometry and topography.^10^ We have previously 3D bioprinted cartilage tissue^11^ derived from chondrocytes and induced pluripotent stem cells (iPSCs).^12^ Clinical trials using iPSCs have been performed worldwide, but clinical translation using iPSCs awaits safety results. Therefore, a step toward earlier clinical use would be to incorporate primary autologous chondrocytes into the 3D constructs. The 3D bioprinting of chondrocytes in nanocellulose/alginate bioink has previously been reported,^13,14^ and 3D bioprinted constructs utilizing these bioinks have been implanted in mice and chondrogenesis observed *in vivo*.^15,16^ Several studies examining 3D bioprinting for cartilage have been published.^17-20^ These studies focus on the development of bioinks and different cartilage repair using animals models in which the defect is artificially induced using drills and other tools.^21^ Both bioprintable biomaterials and chondrocytes have been used in Food and Drug Administration–approved systems. An example of the latter is autologous chondrocyte implantation (ACI), a chondrocyte-based procedure with a clinically acceptable outcome using the patient’s own chondrocytes.^4,22^ An advantage of using chondrocytes instead of, for example, mesenchymal stem cells (MSCs), is that transplanted chondrocytes preferentially differentiate into cartilage, while MSCs by default tend to differentiate into bone.^23^ OA, as a disease that affects the whole joint, is often beyond reach for biological cell repair, and patients with large cartilage defects are mainly scheduled for arthroplasty. Therefore, a new generation of more sophisticated tissue engineering cartilage grafts is needed to treat this more challenging patient population. Our hypothesis is that cartilage lesions caused by injuries or early OA might be treatable with cell therapies using new technologies by 3D bioprinting chondrocyte cells in bioinks with foundation from ACI technology. Therefore, the aim of this study was to use methods preferentially used in the clinic to determine the shape and size of lesions caused by OA and 3D bioprint a mimic of the lesion that would develop into cartilage. In this study, we scanned a human tibial plateau OA defect site using various imaging tools and created a 3D model of the tibia for 3D bioprinting with surplus allograft chondrocytes from a planned ACI procedure.

## Methods

### 3D Imaging of an OA Defect Site

A tibial plateau was retrieved from a patient with OA who had undergone total knee arthroplasty surgery at Sahlgrenska University Hospital, Gothenburg, Sweden. De-identified tissue sampling was performed according to a procedure that was approved by the Ethical Committee in Gothenburg. For preservation, the sample was fixed in 10% formaldehyde for 24 hours, decalcified in 2.5% formic acid for 10 days, and finally washed and stored in phosphate-buffered saline (PBS) solution prior to further analysis. The sample was photographed with a high-resolution 3dMD camera (3dMD Limited, London, UK) using 360 torso, photographing using 7 pods with 3 cameras each, resulting in a total of 21 cameras for imaging.

The sample was then scanned using various clinical 3D imaging tools: MRI, CT, and a 3D scanner.

#### Magnetic Resonance Imaging

**Figure 1.**
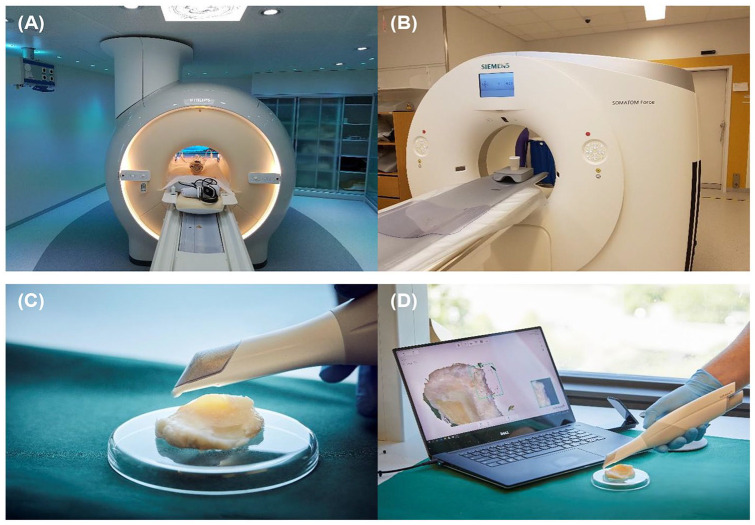
Instrument and experimental setup to generate data for analysis of the tibial plateau. (**A**) Magnetic resonance imaging (MRI) instrumentation setup. (**B**) Computed tomography (CT) instrumental setup. (**C**, **D**) Three-dimensional (3D) scanner experimental setup.

The tibial sample was placed in a plastic beaker with water and scanned with an MR Philips Ingenia 3 Tesla Instrument using the following parameters: Coil: wrist coil, scan type: 3D, technique: SE (spin echo), TE (echo time): shortest, flip angle: 90°, TR (repetition time): 1500 ms FOV (field of view) FH (foot-head): 180 mm; AP (anterior-posterior): 180 mm; RL (right-left): 100 mm. The experimental setup is shown in **Figure 1A**.

#### Computed Tomography

The tibial sample was analyzed using CT: Siemens SOMATOM Force. Double energy (DE), dual energy CT using the following parameters: total: 1781 mA·s; total DLP: 56 mGy·cm; scan 1: 120 kV, mA·s/ref: 19 mA; CTDiVol* (mGy): 0.07 L; DLP (mGy·cm): 1.5; Ti: 2.3 seconds; cSL: 0.6 mm; scan 2: 100 kV, mA·s/ref: 19 mA; CTDiVol* (mGy): 0.04 L; DLP (mGy·cm): 1.0; Ti: 2.5 seconds; cSL: 0.6 mm; scan 3A: 70 kV; mA·s/ref: 200 mA; scan 3B: Sn150 kV; mA·s/ref: 50 mA. CTDiVol* (mGy): 4.66 L; DLP (mGy·cm): 53.9; Ti: 2.5 s; cSL: 0.6 mm. The experimental setup is shown in **Figure 1B**.

#### 3D Scanner

The sample was scanned using a hand scanner (TRIOS 3 wireless, 3Shape A/S, Copenhagen, Denmark) on both sides, and the 2 halves were saved in separate stereographic format (stl.) files. **Figure 1C** and **D** shows the experimental setup for scanning the tibia. MeshLab Version v2016.12 software was used to merge the 2 surface models into 1 stl. file.^24^ The total scanning time was approximately 2 to 3 minutes.

### Generation of a CAD Model of an OA Defect Site

To create a CAD model of the OA defect, the stl. file was loaded into CATIA V5 (Dassault Systémes, Paris, France). The tibial plateau image was subdivided into a focus area that included the OA defect. From this area, a surface model was created. Within this surface model, the OA defect was sketched out, and a new surface was created. This surface was copied and translated 0.25 mm in an appropriate direction to approximately level out with the healthy cartilage. By creating boundaries of the OA defect and the translated surface, a surface joining the OA defect and translated surface was created. By joining these 3 surfaces (OA defect, translated surface, and joining surface), a closed solid volume was created, which was then saved as an stl. file, which was used for the 3D print model using PA12 material and an EOS P760 printer from EOS.

### Fabrication of Bioinks

Nanocellulose/alginate bioink with a composition of 80% nanocellulose (NFC) and 20% alginate (A) was prepared as described previously.^13^ CELLINK start and CELLINK bioink from CELLINK AB, Sweden were used for *in situ* bioprinting.

### 3D Bioprinting

Grid constructs of the mold were designed using slic3r Version 1.3.0-dev, and the constructs were bioprinted using bioink 80:20 NFC:A. The printing was performed in a 3D bioprinter, INKREDIBLE, from CELLINK AB, Sweden, in a LAF (laminar flow hood) bench in a clean room. *In situ* 3D bioprinting was performed with a BioX 3D bioprinter from CELLINK AB in Sweden.

### Isolation of Human Chondrocytes from Articular Cartilage

Human chondrocytes were prepared at a GMP (Good Manufacturing Practice) facility. From 3 anonymized donors who had consented to donate cartilage for research, cells were isolated from cartilage by cutting them into pieces followed by rinsing with PBS. The tissue was digested using 0.1% trypsin for 30 minutes, followed by 0.1% hyaluronidase for 60 minutes and then 0.1% collagenase type II overnight at 37°C. The enzymatic digestion was quenched with human serum, and the cell suspension was filtered through a sterile cell strainer (pore size 40 µm), centrifuged, and seeded in chondrocyte medium: Dulbecco’s modified Eagle medium (DMEM)/F12 with 10% human serum, 2 mM l-glutamine and 0.1 g/L l-ascorbic acid. The isolated chondrocytes were expanded at 37°C and 90% humidity in 5% CO_2_, never frozen and used at passage 1. Chondrocytes were passaged using 0.1% trypsin. All human material was surplus from anonymized patients who provided written consent for use in research, and the decoded material used as chondrocytes or the tibial plateau cannot be traced back to the donor. Ethical permission was approved by “The Regional Ethical Review Board in Gothenburg,” reference number: 713-17 (www.epn.se).

### 3D Bioprinting with Chondrocytes

The concentration of chondrocytes was 20 million per milliliter (80:20 NFC:A bioink). The 3D bioprinting was performed at room temperature in a clean room with a 3D bioprinter (INKREDIBLE). Filtered air was used to reduce the risk of contamination, and prior to the 3D bioprinting, the apparatus was sterilized using 70% ethanol. Grid constructs of the mold were designed using slic3r Version 1.3.0-dev, and the constructs were bioprinted using bioink 80:20 NFC:A. The printing pressure during printing was 5 kPa for 80:20 NFC:A 3D bioprints. The 3D bioprints with chondrocytes were bioprinted with a 410-µm nozzle.

Directly after 3D printing in a tissue well, the constructs were crosslinked for 5 minutes in 100 mM CaCl_2_. Finally, the crosslinked constructs were rinsed in culture medium. The culture medium was replaced with fresh medium, and the constructs were placed in an incubator at 37°C and 5% CO_2_ for 2 days to recover before differentiation (see next section) for 2 weeks.

### Chondrogenic Differentiation

Control chondrocytes (200,000/well) were centrifuged to form pellets in a 96-well round-bottom low-attachment plate. The plate was centrifuged for 5 minutes at 500 × *g* to form pellets, with one in each 96 well to form a micro tissue following differentiation for at least 2 weeks. Then, the 3D bioprint with and without chondrocytes and the control chondrocyte micro tissue pellets were induced to differentiate by replacing the defined medium (DEF) with chondrogenic differentiation medium, consisting of DMEM-high-glucose (high-glucose DMEM; PAA Laboratories) supplemented with 5.0 mg/mL linoleic acid solution (Sigma-Aldrich), 1× ITS-G premix (6.25 mg/mL insulin, 6.25 mg/mL transferrin, 6.25 ng/mL selenous acid; Life Technologies), 1.0 mg/mL human serum albumin (Equitech-Bio, Kerrville, TX, USA), 10 ng/mL TGFβ1, 10 ng/mL TGFβ3, 100 nM dexamethasone (Sigma-Aldrich), 80 nM ascorbic acid 2 phosphate (Sigma-Aldrich), and 1× penicillin/streptomycin (PEST; PAA Laboratories). The medium was changed 3 times a week. The 3D bioprints and control chondrogenic pellets were harvested after 14 days for histological and reverse transcription–polymerase chain reaction (RT-PCR) analysis. The cell number and viability were counted before and after 3D bioprinting using nucleocounter NC-200TM in Via1-CasettesTM (ChemoMetec, Denmark).

### Histological Preparations

Samples were rinsed twice with PBS containing CaCl_2_ before fixation with Histofix (5% paraformaldehyde; HistoLab Products AB, Gothenburg, Sweden), also supplemented with CaCl_2_ (to prevent the prints from falling apart), overnight. Next, the samples were rinsed twice with PBS before being stored in 70% ethanol for transport to HistoLab (Gothenburg, Sweden) for paraffin embedding, slicing (10-µm sections), and staining with Alcian blue and van Gieson’s dye for glycosaminoglycans. An upright Nikon Eclipse 90i microscope was used to obtain images of the histology sections.

### Microscopy

Microscopy images were obtained of the samples with a wide-field fluorescence confocal microscope; bright-field images were obtained using a Nikon Eclipse Ti-U camera.

### RNA Extraction and RT-PCR

Samples were frozen at −80°C after 2 weeks of differentiation for RNA extraction. Lysis of the construct was performed with RLT buffer from Qiagen Mini-Kit and Matrix Lysis D (MP Biologics), which was shaken at 25 Hz for 2 minutes on a Qiagen Tissue Lyser. The lysate was then used for RNA extraction following the standard protocol from the Qiagen Mini Kit. The RNA concentration and quality were obtained immediately after extraction using the NanoDrop 2000 (Thermo Fisher). For cDNA synthesis and quantitative RT-PCR, all reagents, instruments, and software were purchased from Applied Biosystems (Life Technologies). The cDNA was prepared from total RNA using a High-Capacity cDNA Reverse Transcriptase Kit with random hexamers and RNase Inhibitor on a 2720 Thermal Cycler. All samples were analyzed in biological triplicates and thereafter duplicates on the 7900HT instrument using TaqMan Gene Expression Master Mix. The following human TaqMan gene expression assays were used: COL2A1, splice variant type B (Hs01064869_m1), type A, (Hs00156568_m1) and ACAN (Hs00153936_m1). CREBBP (Hs00231733_m1) was used as a reference gene. All samples were treated with RNase-Free DNase (Qiagen GmbH, Hilden, Germany) to avoid genomic DNA contamination. The fold change for each sample was calculated using the 2-ΔΔCT method,^25,26^ and the expression level was calculated relative to an in-house calibrator. The Student *t* test was used to calculate significance with 3 replicates, and *P* < 0.05 was considered statistically significant. Data are presented as the mean values ± standard deviation (SD).

### *In Situ* Bioprinting

The BioX 3D bioprinter (Cellink AB, Sweden) along with the generated CAD file of the cartilage filling were used. The precision in *xy* and *z* was good, and to calibrate the printing of the reparation on the damaged knee, the stl file of the repair was flattened and remarkable points of the repair were placed on the 3D plastic printed of the damaged knee. Without turning it, remarkable points of the 3D plastic printed damaged knee were then placed on a 96-well plate lid and marked with a pen. While printing, the remarkable points of the real damage were placed on the prepared marks of the 96-well plate. Z calibration was then performed in the middle of the damaged construct.

## Results

### 3D Imaging

**Figure 2.**
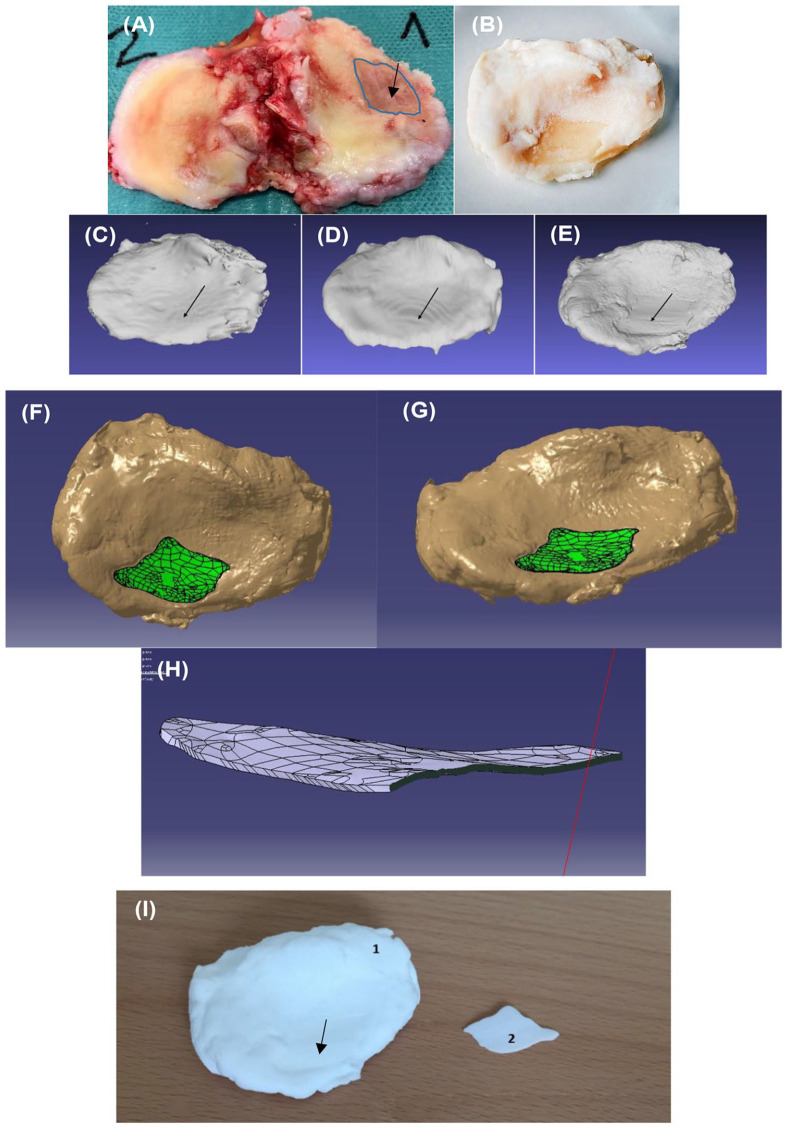
(**A**) Tibial plateau obtained from knee prosthesis surgery with an osteoarthritic (OA) defect (arrow). (**B**) Tibial plateau (1) after fixation and decalcification showing an OA defect (arrow). (3dMD camera). (**C**) CAD model of the tibial plateau obtained from magnetic resonance imaging (MRI), (**D**) CAD model of the tibial plateau obtained from computed tomography (CT), and (**E**) computer-aided design (CAD) model of tibial plateau obtained from hand scanning data showing the OA defect (arrow). (**F**, **G**) CAD model obtained from hand scanning data of the tibial plateau with filling of the defect (green). (**H**) CAD model obtained from hand scanning data of the OA defect filling. (**I**) 3D printed model of the tibial plateau (1) and defect (2).

To retrieve the medial condyle with OA lesion that can be bioprinted into *in situ*, whole tibial plateau was collected directly after total knee arthroplasty surgery. Photograph of tibial plateau taken shows that the cartilage appears healthy without any defects or damaged area on the lateral condyle (left side), except the area close to the center of the tibia. In contrast, there is a large and deep cartilage defect on the medial condyle (right side, marked in blue) (**Fig. 2A**). The condyle with OA-caused damage cartilage was cut out and further processed to preserve the structure included fixation and decalcification, as described in the experimental section (**Fig. 2B**). To generate a 3D model of the OA defect, 3 different scanning 3D imaging tools were used with the experimental setup shown in **Figure 1**. The 3 scanner equipment that we tested herein are (1) MRI, (2) CT, both used in orthopedic clinics, and (3) a 3D portable scanner, which has recently been introduced in odontology clinics.

**Figure 3.**
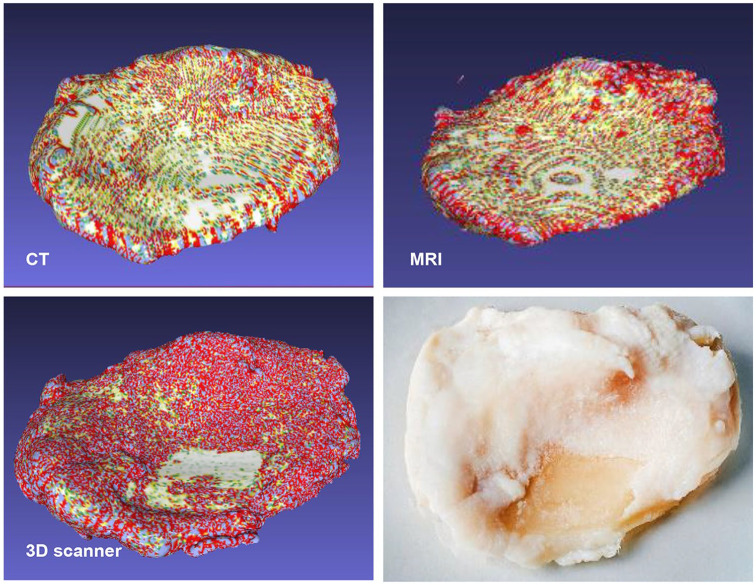
Gaussian curvature analysis of 3D models of the tibial plateau generated from scanning (**A**) computed tomography (CT), (**B**) magnetic resonance imaging (MRI), and (**C**) 3D scanning of (**D**) the tibial plateau. Red color indicates a positive Gaussian curvature, blue color indicates a negative Gaussian curvature and green color indicates that the Gaussian curvature approaches zero.

The decalcified tibia with an OA defect taken with a high-resolution camera are shown in **Figure 2B**. After scanning a 3D model of the dissected tibia (**Fig. 2B**) was constructed using MRI (**Fig. 2C**) or CT (**Fig. 2D**) or 3D optical hand scanner (**Fig. 2E**) of the same tibia. After scanning using the 3D scanner, the OA cartilage defect was clearly visible (arrow in **Fig. 2E**). To further compare the different scanning techniques, we used the slicer program version 4.8.1 (www.slicer.org)^27^ and the results also shows that the 3D scanner had the highest resolution, with 2.77 times more points than CT and 3.27 times more points than MRI (**Table 1**). Gaussian analysis of the generated 3D models of the tibial plateau obtained from scanning using MRI, CT, or 3D scanning also indicates that the 3D scanner generated a 3D model with more topological variance (**Fig. 3**). The different scanning techniques were compared, and the estimated difference between the volume of the tibial plateau, calculated by the Archimedes principle and the volume of the corresponding 3D models obtained after scanning with MRI, CT, or 3D scanning, was calculated (**Table 1**). The 3D scanner also provided the most accurate estimation of the volume of the tibial plateau compared with when immersing the tibia in water, with a difference of approximately 4% to 5%. The 3D models obtained after scanning the sample using MRI or CT show a large underestimation of the volume of the tibia by −37% and −19%, respectively (**Table 1**). The cartilage defect area was only detected with 3D scanner (**Fig. 3**, **Table 1**). We also tested micro CT (µCT) that is known to have better resolution. Scanning using µCT obtained a surface area of the defect of 218.4 mm^2^ (**Table 1**) and an average thickness of the cartilage for this condyle of 1.98 mm. The damage reached down to the bone, which resulted in an estimated defect volume from µCT of 218.4 mm^2^ × 1.98 mm = 431 mm^3^. Since the cartilage defect area from µCT differ as much as 44% from 3D scanner, we measured the visible defect in the photograph (**Fig. 2B**, **Table 1**) by using Image J to calculate the area by inclusion of a scale in the photo. We also estimated the area by drawing by hand on overhead film by placing it over the damage decalcified tibia and lay on top on millimeter graph paper. Our conclusion is that the 3D scanner was the most accurate.

**Table 1.** Scanning Precision MRI, CT, and 3D Scanner versus the Volume of the Tibial Plateau Calculated by Immersing the Tibial Plateau in Water.^a^

Scanning Technique	Volume Tibia (mm^3^)	Difference (%)	Surface Area OA Lesion (mm^2^)	Difference (%)
Tibial plateau calculated using the Archimedes principle	17143.84	0		
Area calculated from photo using ImageJ			390.3	0
MRI	10733.53	−37.39133123	UD	−100
CT	13864.65	−19.12751169	UD	−100
3D scanner	17908.6	4.460844245	393.09	0.7
µCT	NA		218.4	−44
Surface Tibia	Number of Points		Number of Cells	
MRI	65,216		129,940	
CT	77,034		154,060	
3D scanner	213,626		423,767	

OA = osteoarthritis; MRI = magnetic resonance imaging; CT = computed tomography; µCT = micro computed tomography; UD = undetectable; NA = not analyzed.

a Scanning precision MRI, CT 3D scanner and µCT versus surface of OA lesion calculated by photo and Image J.

### Development of the 3D Model of the OA Defect

For the reverse engineering process, we selected the stl file obtained from the 3D scanner. The digital repair (green) of the OA defect in the model of the tibial plateau was constructed using CATIA V5 (Dassault Systémes, Paris, France) software to generate the filling (**Fig. 2F** and **G**). The generated stl file of the 3D model of the cartilage defect is visulized in **Figure 2H**. The obtained information was also used to 3D print a model of (1) the tibial plateau including the OA defect and (2) the defect, which can be used as the template for the filling (**Fig. 2I**).

**Figure 4.**
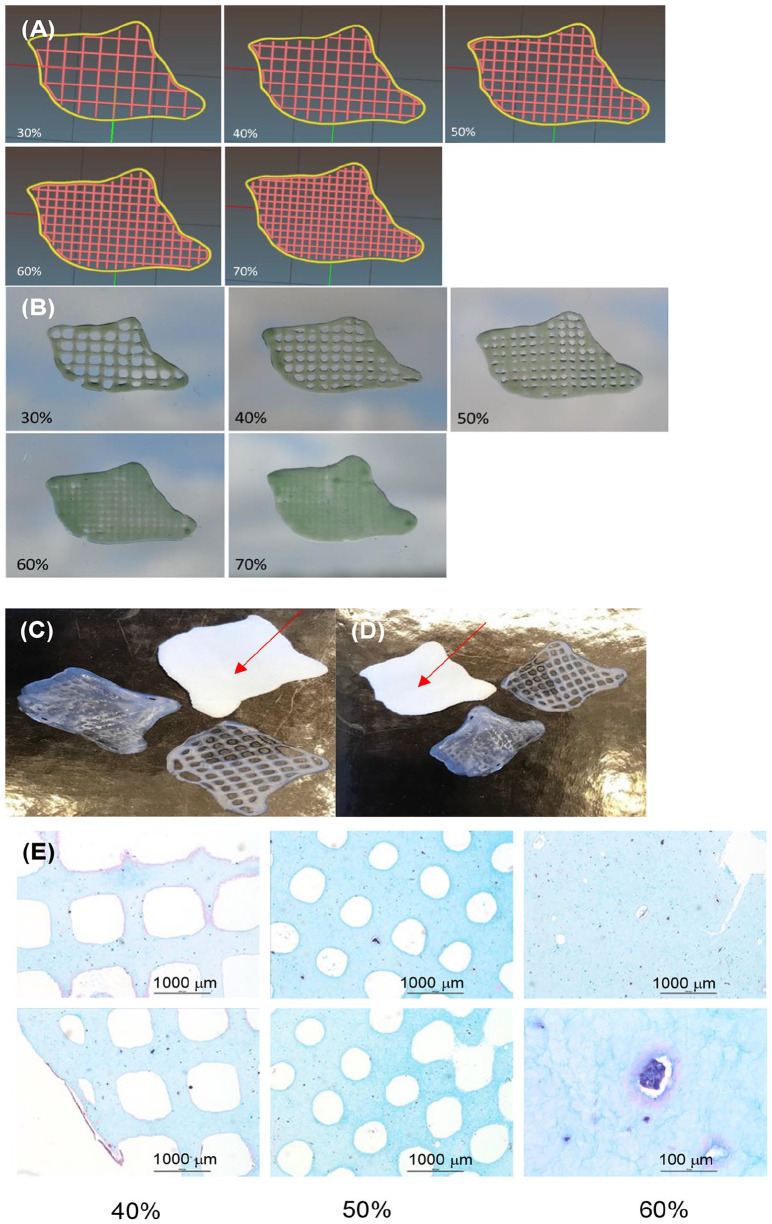
(**A**) Computer-aided design (CAD) models of constructs with different infills (30% to 70%) and corresponding (**B**) optical images of 80:20 NFC:A printed constructs with different infills (30% to 70%). (**C**, **D**) 3D bioprinted constructs and 3D printed model of the filling (red arrow). (**E**) 3D bioprinted chondrocytes in 80:20 NFC:A with different infills. Scale bars are 1000 µm and 100 µm, as indicated.

The G-code (obtained from the stl file) was used to control the bioprinter, and a test print of the constructs in 80:20 NFC:A bioink without cells was performed obtaining a grid-construct of the CAD model, (**Fig. 4A-C**). It was also possible to print these constructs with different infills (30%, 40%, 50%, 60%, and 70%) (**Fig. 4B**). A good match was obtained between the bioprinted constructs and the 3D printed model, (**Fig. 4C** and **D**). Histological sections of bioprints stained with Alcian Blue van Gieson using 40%, 50%, and 60% infills with chondrocytes, following chondrogenic differentiation for 2 weeks (**Fig. 4E**).

### 3D Bioprinting with Cells

**Figure 5.**
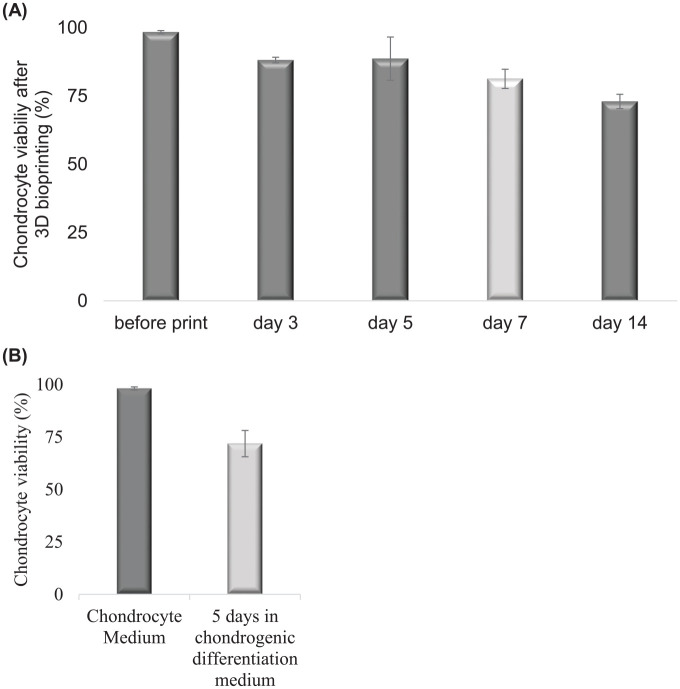
Cell viability at different time points for (**A**) 3D bioprinted chondrocytes differentiated in 80:20 NFC:A bioink for 2 weeks. (**B**) Cell viability of control chondrocytes in the 2D monolayer in chondrocyte medium or chondrogenic differentiation medium for 5 days, comparable to day 7 in (**A**), light gray.

**Figure 6.**
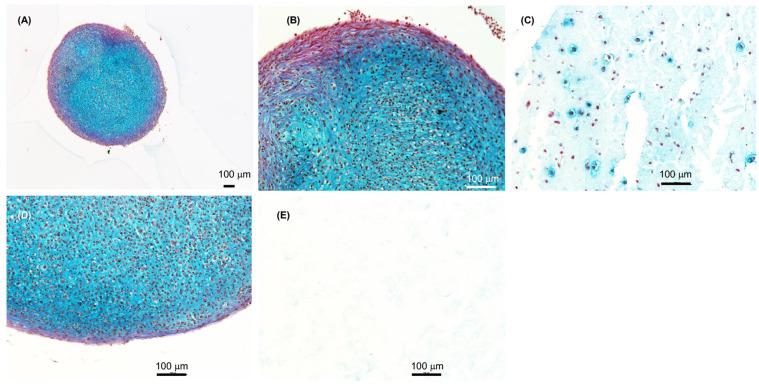
Histology sections of chondrocytes differentiated for 2 weeks. (**A-C**) 3D bioprinted primary chondrocytes in 80:20 NFC:A bioink followed by differentiation for 2 weeks. Micro tissue structures similar to pellets can also be seen in the 3D prints. (**D**) Control primary chondrocytes differentiated as micromass pellets for 2 weeks. (**E**) Control print 80:20 NFC:A bioink with no cells. Scale bars, 100 µm.

The chondrocyte-cell viability remained high after 3D bioprinting, with a slight decrease at day 3 (from 98% to 88%, *P* < 0.05) after printing as well as day 14 (from day 7, 81% to 72%, *P* < 0.05), the decrease at day 5 and 7 is not significant. The cell viability after 3D bioprinting was measured at different time points, explicitly before printing and at day 3, day 5, day 7, and day 14 are shown (**Fig. 5A**). This decrease in cell viability was in the same range as observed during control chondrogenic differentiation in pellets in 3D, and the difference was not significant (**Fig. 5A** compared with **5B**, day 7 in **A** matches day 5 in **B**). Chondrocytes were well distributed in the 3D prints (**Fig. 6A-C**) and our results demonstrates that cartilage tissue is formed after 3D bioprinting and the chondrocyte clusters produced extracellular matrix (ECM) (dark blue) (**Fig. 6A** and **B**). The histology sections were stained with Alcian blue and van Gieson’s dye after differentiation of the 3D bioprinted chondrocytes for 2 weeks in the presence of chondrogenic medium in 80:20 NFC:A bioink. Control cartilage tissue formation (dark blue) of histology sections of micromass pellets formed from control chondrocytes (the same batch of primary chondrocytes that was used for 3D bioprinting) (**Fig. 6D**). The negative control, and as expected, shows no cartilage tissue formation; nor were cells detected in the histology section of 3D bioprints without cells (**Fig. 6E**).

**Figure 7.**
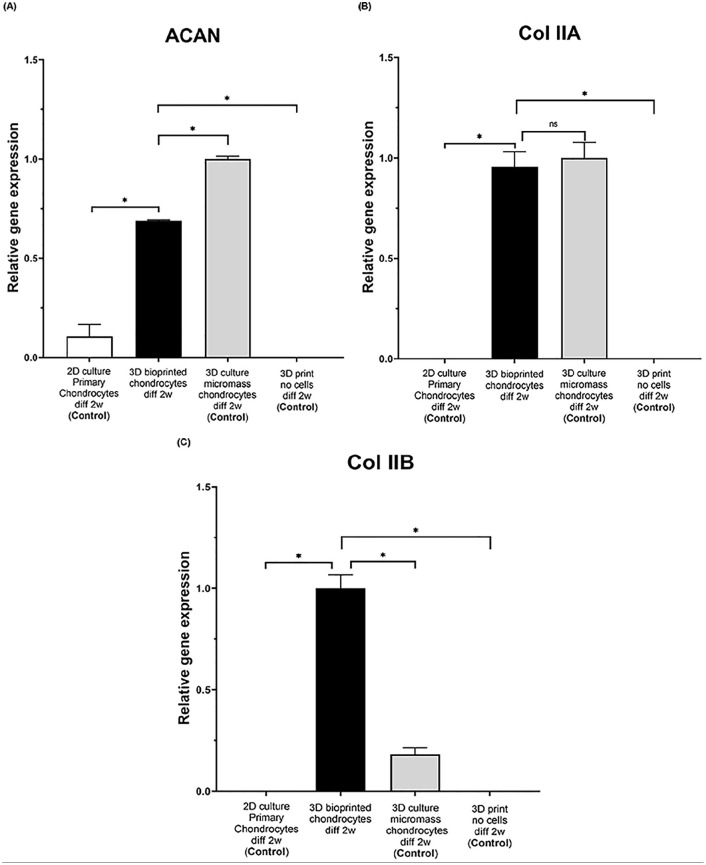
Aggrecan (ACAN) and collagen type II procollagen (COL 2A1 splice variants type IIA or type IIB) expression in 2D culture of primary chondrocytes, 3D bioprinted chondrocytes in 80:20 NFC:A bioink, no cell 3D printed control and micromass chondrocytes (all samples followed by chondrogenic differentiation for 2 weeks), analyzed by RT-PCR analysis. (**A**) 3D bioprinted chondrocytes show high levels of ACAN compared with nonprinted primary chondrocytes (*P < 0.05); micromass chondrocytes show slightly higher levels of ACAN compared with 3D bioprinted chondrocytes (*P < 0.05). (**B**) Collagen II type A (Col IIA type A). (**C**) Collagen II splice variant type B (COL IIA type B) is expressed at high levels in 3D bioprinted chondrocytes compared with primary chondrocytes and micromass chondrocytes (*P < 0.05). Each bar represents one condition (n = 3), as indicated on the *x*-axis, and the expression is normalized to CREBBP and to the highest expression for each gene in each separate condition. Student’s *t* test was used to determine statistical significance between the different conditions and considered significant if *P* < 0.05, as indicated by *.

After 2 weeks of differentiation of 3D bioprinted chondrocytes, expression of aggrecan (ACAN) and collagen type II (both splice variant A and the more mature version B that are predominately found in native cartilage) were analyzed (and as controls; 2D culture of chondrocytes (primary chondrocytes), 3D micromass pellets of chondrocytes and 3D print no cells, all differentiated for 2 weeks) (**Fig. 7A-C** and Supplemental Figure 1). The ACAN gene expression analyzed by quantitative RT-PCR (qRT-PCR) was significantly higher for the 3D bioprinted chondrocytes (average 6-fold increase) and the micromass chondrocytes (average 9-fold increase) compared with primary chondrocytes, (**Fig. 7A**). Furthermore, the 3D bioprinted chondrocytes had slight but statistically significant lower ACAN gene expression (average 1.5-fold decrease, *P* = 3 × 10^−6^) than the control chondrocyte-derived micromass pellets (**Fig. 7A**).

Collagen type II splice variant A showed significantly higher gene expression in 3D bioprinted chondrocytes and micromass chondrocytes compared with primary chondrocytes (**Fig. 7B**). The 3D bioprinted chondrocytes showed statistically significant higher collagen type IIB expression compared with control micromass chondrocytes (average 5-fold increase, *P* = 5 × 10^−5^) (**Fig. 7C**).

### *In Situ* Bioprinting

**Figure 8.**
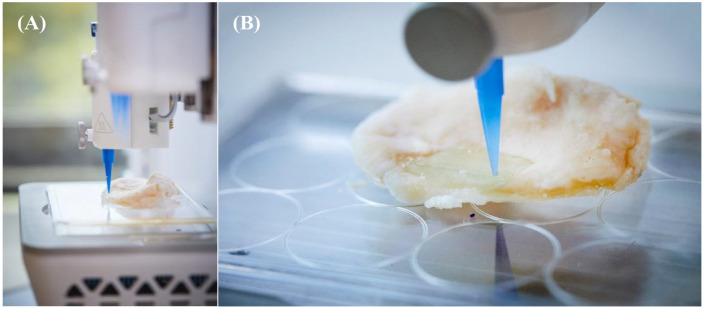
(**A**) *In situ* bioprinting setup using a bioprinter. (**B**) *In situ* bioprinting into a cartilage defect in a tibial plateau of an osteoarthritic patient donated after total knee arthroplasty surgery.

For the final step in the *in situ* 3D bioprinting approach that could be used during surgery, chondrocyte containing ink was 3D bioprinted directly into the OA defect of the donated tibial plateau. The printer setup (**Fig. 8A**) and a snapshot of the printing process are shown (**Fig. 8B**). The precision in *xy* and *z* made it possible to completely fill the defect with a layer of bioink.

## Discussion

For more than 30 years, local cartilage traumatic lesions have been successfully treated by ACI.^4,22^ In this study, we developed a workflow and setup to examine how 3D imaging, 3D bioprinting technology and chondrocytes from ACI could be used for patient-specific cartilage repair. First, we showed how the CAD model of around 400 mm^3^ (2 cm × 2 cm × 1 mm) anatomical OA defect could be constructed based on images acquired with imaging tools that are available in the clinic. With clinical tools such as CT, MRI, and other 3D imaging scanning, exact visualization of the cartilage defects can be achieved and the images converted into patient-specific 3D CAD models. Using such a digital model, healthy twin cartilage copies of the diseased and surgically debrided area can be produced by 3D bioprinting with chondrogenic cells in bioink. As proof of concept, by 3D scanning of a tibial plateau, CAD models were created of an OA defect using special CAD software. Furthermore, the 3D scanning portable instrument (3D scanner), which has been recently introduced for odontology, was found here to be superior with regard to time and resolution compared with 3D imaging tools such as MRI and CT, and it was the only method (except for µCT) that was able to visualize the actual cartilage OA defect. Therefore, we suggest that the 3D scanner is the best choice for scanning during open surgery, which is an undesirable option why further technical developments are required, for example, scaling down, to be used in future arthroscopic tissue engineering procedures. The CAD model, which was created from the image obtained with the 3D scanner, was used to generate a G-code, which controlled the 3D bioprinter. Using this setup, it was possible to 3D bioprint directly into the cartilage lesion area and fill the large OA defect with bioink-containing cells. This process could be performed after adaptation as an arthroscopic procedure.

In addition, the 3D bioprinted chondrocytes produced extracellular matrix comparable to native cartilage after being 3D bioprinted into the cartilage defect site. The 3D bioprinted chondrocytes produced aggrecan (ACAN) and collagen II (Col II type B) characteristic of native cartilage after two weeks of differentiation in cell culture medium. Type II collagen can be synthesized in two forms, type A or type B, generated by alternative splicing of the precursor mRNA.^28^ Type IIA procollagen contains a cysteine-rich domain in the NH_2_ terminus of the propeptide in exon 2, and type IIB lacks this domain. Type IIA is found in precartilage, and noncartilage epithelial and mesenchymal cells carrying the type IIB procollagen variant are characteristic of chondrocytes found in cartilage. Collagen type II is normally low or nondetectable in primary chondrocytes after growth in culture. In the 3D bioprinted chondrocytes, high levels of collagen type II type B were observed, indicating that after 2 weeks of differentiation, the 3D bioprinted chondrocytes produced collagen type II characteristic of native cartilage. This result indicates that the 3D printing procedure and the ink used herein support chondrocytes to produce extracellular matrix that is characteristic of native cartilage. Furthermore, in support of that the histological sections of the 3D prints shows area of formed cartilage tissue. To form a tissue a large number of cells are required,^11^ and as viability is slightly decreased in the prints, more cells in the prints nutrients become restricted. Here we used 20 million chondrocytes per milliliter, which were successful for tissue generation, but this number needs to be further optimized for tissue formation in the whole print. However, the alginate containing ink has previously shown to fall apart without double charged ions but *in vivo* the ion content is likely to be sufficient for it to be stable and the chondrocytes are anticipated to continue to form cartilage and replace alginate, and in support nasal chondrocytes in NFC:A ink is stable for 60 days.^16^

Together, ACAN and collagen II form a major structural component of articular cartilage.^29,30^ Subsequently, the results presented in this pilot study show that 3D bioprinting of chondrocytes might be used for cartilage lesion repair with potentially exact lesion filling related to the CAD modeling. OA develops slowly and have passed several stages like pre-OA and early OA until it ends up in a total joint destruction. Local cartilage repair has previously shown to halt and slow down such a destructive process.^31^ Therefore, it seems tempting to debride such diseased areas for a local repair. Until recently, reports on tissue engineered constructs mostly lack the normal spatial complexity in cell types and tissue organization, a fact that may explain a relatively limited success to date. That is why there is now an increased interest in bioprinting technologies as it is possible with a cell layer technique to produce a more hyaline-like repair. Widespread OA is successfully treated with total arthroplasties. However, arthroplasties have a limited lifetime of 15 to 20 years, when aseptic loosening of the implant may occur, making young OA patients more suitable for biological resurfacing.^32^ It is currently not possible to resurface osteoarthritic joints by cartilage tissue engineering, underscoring the potential importance of early interventions. Clinically, as treatment alternatives for early OA, one may transfer this pilot technique to produce a healthy 3D printed implant that is customized based on the patient’s joint anatomy and the area of damaged OA cartilage, using details obtained from more specialized MRI than tested herein or arthroscopic scans. Patients with early OA lesions would be offered a customized tailor-made 3D printed construct targeting the damaged OA cartilage and, prior to implantation, the cartilage area to be resected could be outlined for surgery. The 3D printed implants with genetically modified chondrocytes may be designed to stop or delay local OA lesions to develop into widespread OA. To transfer the presented technology to the clinic different repair technology may be used: either direct *in vivo* chondrogeneic cell printing with a miniature autoscopic bioprinter into the exact debrided lesion area or with 3D printing production of an osteochondral implant produced *in vitro* for later implantation. Cell choice could be autologous chondrocytes/mesenchymal stem cells or allogeneic chondrogeneic cells. For a direct *in vivo* 3D printing of cells, the scaffold choice may vary. An ink being liquid at room temperature but turns solid at body temperature at lesion site after 3D articular printing is an interesting choice, or chemically modified with drug to combat OA. A treatment of pre-OA and early OA lesion will then become part of strategies for joint preservation.

The 3D tissue models can be printed from all types of volumetric image data sets with sufficient contrast to differentiate between tissues. CT for 3D scanning is commonly used because of easy handling of the image postprocessing. The negative exposition to radiation when using CT is avoided when using MRI or the 3D scanner. Surprisingly, we found that the CT or MRI used in the clinic lacked sensitivity to detect the OA lesion, while earlier laboratory studies detected artificially made drilled defects. Therefore, we conclude that an authentic OA lesion cannot always be detected by CT or MRI. Early interventions, treating local chondral and osteochondral defects on demand with allogeneic chondrogenic cells bioink-printed directly into the defect area through an arthroscopic 3D scan of the injured area, is our future visionary goal.

The limitation of this study is that only one tibial explant was used and that the mechanical properties of the chondrocyte prints have not been tested to withstand compression forces of the femur in a joint. In the future, and since only the invasive 3D scanning was successful, the possibility of 3D arthroscopic scanning combined with arthroscopic printing should be evaluated. Early interventions treating localized “pre-OA” lesions with healthy chondrogeneic repair cells might be a future alternative for joint preservation. Last, *in situ* printing can potentially reduce cost, since the growth of cartilage in a bioreactor would be omitted.

## Conclusion

This study demonstrates that to obtain a 3D model of an OA defect to be used as a template for 3D printing of chondrogenic cells in bioink, the defect area is best visualized by a handheld 3D scanner, while CT and MRI are not as precise. Micro CT had higher resolution than CT but underestimated the lesion area. It was also shown that it is feasible, using 3D modeling of an osteoarthritic cartilage lesion, to create a filling of the defect via 3D bioprinting of human primary chondrocytes containing bioink. The cell viability remained high after bioink printing, with a slight decrease at day 14 (72%), as observed following differentiation. At that time, high levels of a mature version of collagen II (Col IIA type B) and aggrecan (ACAN) could also be found, indicating that this workflow supports differentiation toward native articular cartilage.

## Supplemental Material

Supplemental_figure – Supplemental material for Collagen 2A Type B Induction after 3D Bioprinting Chondrocytes *In Situ* into Osteoarthritic Chondral Tibial Lesion Click here for additional data file. Supplemental material, Supplemental_figure for Collagen 2A Type B Induction after 3D Bioprinting Chondrocytes *In Situ* into Osteoarthritic Chondral Tibial Lesion by Birgitta Gatenholm, Carl Lindahl, Mats Brittberg and Stina Simonsson in CARTILAGE
