# Supplementary figures and images for: Collagen 2A Type B Induction after 3D Bioprinting Chondrocytes In Situ into Osteoarthritic Chondral Tibial Lesion
Source: Cartilage. 2020 Feb 18;13(2 Suppl):1755S–1769S. doi: 10.1177/1947603520903788 (PMC8721610; doi:10.1177/1947603520903788)

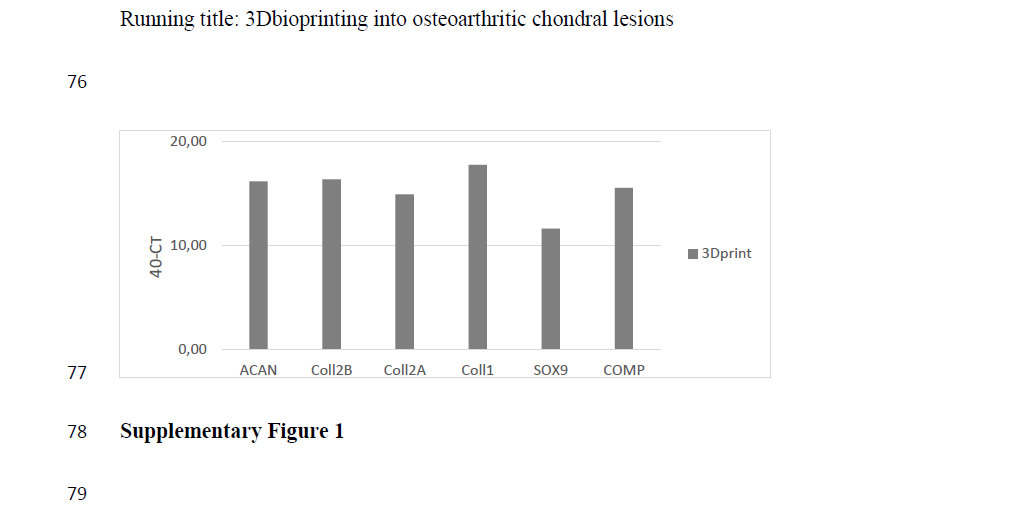

Supplement: Supplemental_figure – Supplemental material for Collagen 2A Type B Induction after 3D Bioprinting Chondrocytes In Situ into Osteoarthritic Chondral Tibial Lesion [file Supplemental_figure.png]
